# OV16 Improves Radiation-Induced Intestinal Injury by Targeting Transglutaminase 2

**DOI:** 10.3390/molecules31111983

**Published:** 2026-06-05

**Authors:** Zhiyan Zhang, He Wang, Yaowen Cui, Yang Lu, Yingying Xu, Min Li, Sifan Liu, Ying Tian, Ziming Xia, Guangjie Zhang, Shuchen Liu

**Affiliations:** 1 College of Life Sciences, Hebei University, Baoding 071002, China; zzyan0911@163.com (Z.Z.); 19832267208@163.com (Y.L.); 2 Academy of Military Medical Sciences, Beijing 100850, China; w543051071@163.com (H.W.); cuiyaowenchn@163.com (Y.C.); 15665648396@163.com (Y.X.); limin82057@163.com (M.L.); 18146539471@163.com (S.L.); tianying1977@126.com (Y.T.); zmxia22@163.com (Z.X.); 3 College of Chemistry and Materials Science, Hebei University, Baoding 071002, China

**Keywords:** OV16, Transglutaminase 2 (TGM2), radiation-induced intestinal injury (RIII), *Orychophragmus violaceus*

## Abstract

Irradiation (IR) can cause intestinal epithelial cell death, damage to crypt stem cells, and mucosal barrier dysfunction, which are the features of radiation-induced intestinal injury (RIII). Our study first discovered a natural small-molecule alkaloid *Orychophragine* D (OV16) with an obvious radiation protection effect. This study aims to investigate the radiation protection effect of OV16 on RIII and its potential molecular mechanism. The results showed that in vitro OV16 exhibited a significant protective effect on an irradiated human small intestinal epithelial cell-6 (HIEC-6) model. Then, transglutaminase 2 (TGM2), which is the key protein for OV16 to exert its anti-RIII protective effect, was identified as a crucial cellular target of OV16 using drug affinity responsive target stability (DARTS), molecular docking, molecular dynamics simulation, cell thermal shift assay (CETSA), and microscale thermophoresis (MST). Moreover, OV16 can upregulate the expression level of TGM2 in the nucleus of HIEC-6. TGM2 can reduce radiation-induced damage by enhancing the proliferation ability and migration ability of HIEC-6 and reducing the generation of γ-H2AX. Collectively, our study first identified TGM2 as a previously unreported therapeutic target for RIII, and provided a future drug design direction for TGM2 allosteric activators using OV16 as a novel molecular template.

## 1. Introduction

Intestinal epithelial cells exhibit rapid proliferation and renewal characteristics and are highly sensitive to ionizing radiation (IR). IR can induce radiation-induced intestinal injury (RIII) [1], characterized by intestinal epithelial cell death, damage to crypt stem cells, and mucosal barrier dysfunction. RIII is a common complication in radiotherapy for abdominal, pelvic, and retroperitoneal tumors. Drug therapy is the most frequently used treatment approach. Developing drugs for the prevention and treatment of RIII and investigating their mechanisms of action are of significant importance.

IR can induce various types of DNA damage, among which DNA double-strand breaks (DSBs) represent the most severe form [2]. DSBs trigger biological effects such as cell cycle arrest, genomic instability, cellular senescence, or apoptosis [3,4,5]. To counteract IR-induced DSBs, most tumor cells abnormally activate DNA damage repair mechanisms, which is a key reason for their radiation resistance and the poor efficacy of radiation therapy (RT). The regulatory role of transglutaminase 2 (TGM2) in this process is increasingly becoming a research focus for deciphering tumor radioresistance [6,7,8,9,10,11].

TGM2 belongs to the transglutaminase family [12]. During radiation-induced DNA repair, nuclear-localized TGM2 plays a central regulatory role [7,10], providing key support for its involvement in tumor radioresistance regulation. Studies reveal that TGM2 can be actively recruited to and accumulates at DSB sites, where it directly interacts with topoisomerase IIα (TopoIIα) to regulate DSB repair. This mechanism is particularly significant in the DNA damage response of lung cancer cells [10]. Conversely, the absence of TGM2 leads to the loss of cells’ ability to efficiently repair DSBs, thereby significantly enhancing their sensitivity to IR. More importantly, dynamic changes in TGM2 translocation to the nucleus were observed following IR treatment. This phenomenon not only confirms the importance of its nuclear function in radiation response but also provides morphological evidence for TGM2’s direct role in regulating DNA repair.

FSK serves as a direct allosteric activator of TGM2, boosting its enzymatic activity and facilitating osteogenic differentiation, thus acting as an innovative small-molecule agent for osteoporosis therapy via targeting TGM2 [13]. Dopamine, a specific substrate of TGM2, mediates the acylation of target proteins to suppress endothelial cell ferroptosis, promote lung regeneration and counteract fibrosis [14]. Cystamine [15] and ZDON [16] are both inhibitors targeting TGM2 transamidase activity; among them, ZDON exerts specific and irreversible inhibitory effects, while cystamine blocks glutamate-induced TGM2 upregulation to inhibit the caspase-3 apoptotic pathway and protect astrocytes from excitotoxic damage, and ZDON eliminates nuclear TGM2-mediated transcriptional silencing to correct abnormal gene expression and exert neuroprotective effects in Huntington’s disease models. Collectively, these well-studied compounds cover three distinct regulatory patterns of TGM2, namely activation, substrate-mediated modification and enzymatic inhibition, establishing a comprehensive reference context and framework for the pharmacological research of TGM2-targeted small molecules. Against this well-defined background, we herein focus on exploring the biological functions and mechanisms of OV16, a novel TGM2-targeted molecule, in the current study.

OV16 is a natural small-molecule alkaloid with a novel structural skeleton isolated from the seeds of *Orychophragmus violaceus*. Activity studies indicate that it possesses significant in vivo and in vitro anti-radiation activity. Cytotoxicity experiments of OV16 on HIEC-6 cells have been conducted in our previous studies. Within the concentration range of 10–200 μM, OV16 did not exert cytotoxic effects on HIEC-6 cells; under irradiation conditions, OV16 could promote cell proliferation and maintain cell survival rates [17]. Similarly, OV16 exerted similar effects in human umbilical vein endothelial cells (HUVECs) [18,19]. Animal experiments showed that OV16 improved the survival rates of mice after irradiation and promoted the recovery of white blood cell, red blood cell, hemoglobin and platelet levels in their peripheral blood. In addition, OV16 could also maintain the integrity of the small intestinal crypt structure and normal villus length after irradiation, thereby exerting a protective effect on mouse intestinal crypt tissues [20]. OV16 also had a significant inhibitory effect on ferroptosis of small intestinal crypt epithelial cells. These findings indicate that OV16 has a good radioprotective effect and possesses great development potential as a novel candidate monomer for the protection against radiation-induced intestinal injury. This study aims to clarify the interaction between OV16 and TGM2, elucidate the molecular mechanism by which OV16 regulates RIII through targeting TGM2, provide experimental evidence for OV16 as a potential RIII preventive drug, and further validate the role of TGM2 in the repair of intestinal radiation damage, providing a new strategy for targeted therapy of RIII.

## 2. Results

### 2.1. DARTS Analysis of the Interacting Proteins of OV16

**Figure 1:**
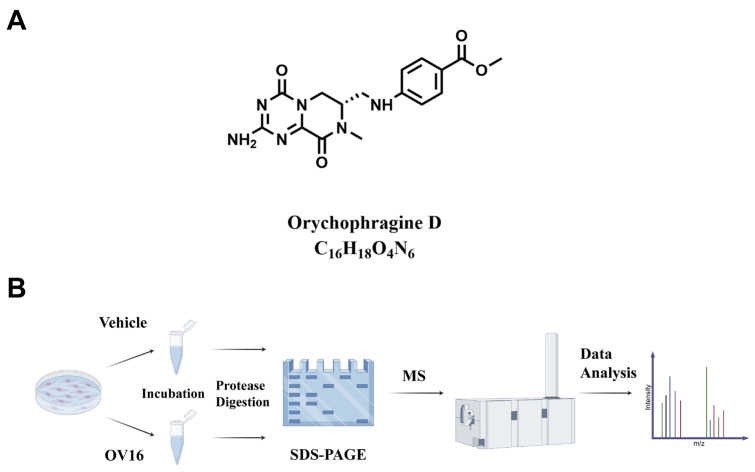
Determination of OV16-targeted protein TGM2: (**A**) structure of OV16; (**B**) flowchart of the DARTS experiment (by Figdraw).

In the research of drug protection mechanisms, drug affinity responsive target stability (DARTS) is primarily used for efficiently identifying molecular targets of candidate drugs. This method detects changes in a target protein’s resistance to proteasome degradation following drug binding, enabling direct screening of potential effector proteins without chemical modification of the drug. It is suitable for target identification in natural products or drugs with complex compositions (Figure 1A,B). Mass spectrometry results from the DARTS assay identified proteins with fold changes exceeding two, which were then ranked based on SUM PEP scores and peptide coverage, revealing TGM2 as an OV16-interacting protein (Table 1). TGM2 belongs to the transglutaminase family, exhibiting Ca^2+^-dependent post-translational modification functions. It catalyzes the formation of covalent bonds between glutamine residues on polypeptide chains and various primary amines, resulting in cross-linked or aminated proteins. TGM2 is also implicated in diverse biological functions including DNA damage, apoptosis, and cell migration [21], and participates in regulating multiple signaling pathways, such as NF-κB signaling [22], EGFR signaling [23], and autophagy [24].

### 2.2. Molecular Docking and Dynamics Analysis of OV16 and TGM2

**Figure 2:**
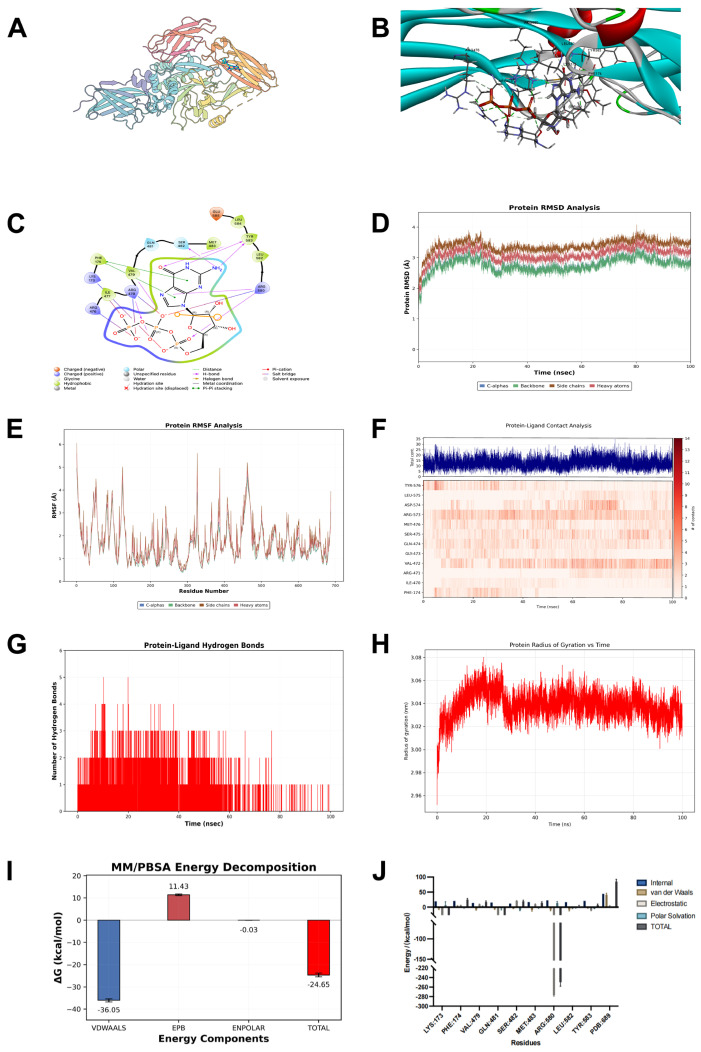
Molecular simulation of OV16 and TGM2. (**A**) Docking of OV16 and TGM2 molecules; (**B**) stable binding between OV16 and TGM2; (**C**) binding interaction between OV16 and TGM2; (**D**) RMSD analysis of OV16 to assess its conformational stability and binding consistency within the TGM2 active site; (**E**) RMSF of TGM2 residues to evaluate local flexibility and identify dynamically perturbed regions upon ligand binding; (**F**) contact analysis between OV16 and TGM2; (**G**) hydrogen bond analysis showing the average number of hydrogen bonds formed between TGM2 and OV16 throughout the MD trajectory; (**H**) *R*_g_ analysis to assess the global compactness and folding behavior of TGM2 during the simulation; (**I**) binding energy (MM/GBSA) calculation of TGM2−OV16 complexes over the simulation time to estimate the binding affinity; (**J**) residue-wise energy contribution analysis to identify key amino acid residues involved in OV16 binding with TGM2.

To further elucidate the interaction mechanism between OV16 and TGM2, molecular docking simulations were performed to predict their binding mode. The docking results demonstrated that OV16 is favorably accommodated within the binding pocket of TGM2, establishing a robust interaction network (Figure 2A,B). Specifically, the 2D interaction diagram (Figure 2C) revealed that OV16 forms critical hydrogen bonds with key residues, including LYS173, ARG580, and TYR583. Furthermore, the highly electronegative phosphate groups of OV16 engage in strong electrostatic interactions with positively charged residues, such as ARG476 and ARG478, further anchoring the ligand within the active site. While molecular docking provides valuable insights into static binding modes, evaluating the dynamic stability of the complex under physiological conditions is essential.

To validate the structural stability of the predicted OV16–TGM2 complex, a 100 ns molecular dynamics (MD) simulation was conducted. Root mean square deviation (RMSD) was calculated to measure structural variations relative to the initial conformation. For a comprehensive dynamic characterization, the RMSD analysis was divided into four components: C-alpha atoms, backbone, side chains, and heavy atoms. All RMSD trajectories exhibited an initial increase during the first 20 ns, reflecting the structural relaxation required for the system to reach thermodynamic equilibrium. Following this 20 ns period, all curves converged to a stable plateau, confirming that the overall protein structure had achieved equilibrium (Figure 2D). Root mean square fluctuation (RMSF) analysis was employed to evaluate the mobility of individual amino acid residues, where low RMSF values correspond to structurally rigid cores and high values indicate flexible segments. Residues within the primary ligand-binding region exhibited moderate-to-low RMSF values, indicating that ligand binding restricts residue mobility and stabilizes the conformation of the binding pocket (Figure 2E). The total number of contacts between OV16 and TGM2 remained consistently high throughout the simulation. Contact heatmaps identified core anchoring residues (e.g., TYR576 and ARG573) and auxiliary residues (e.g., MET476) (Figure 2F), which collectively contribute to the stability and specificity of the OV16–TGM2 interaction. Moreover, MD trajectories revealed the persistent formation of one to five hydrogen bonds between the two molecules (Figure 2G). Although these hydrogen bonds undergo dynamic breaking and reforming, their sustained presence strongly supports binding stability, indicating that the ligand is firmly retained in the pocket via a stable hydrogen bond network. The radius of gyration (*R*_g_) was calculated to assess global structural compactness. Following a brief equilibration phase, the *R*_g_ value stabilized at approximately 3.04 nm throughout the remainder of the simulation, lacking any persistent upward or downward trend. This confirms that the protein maintained its native compact folded state without undergoing structural expansion or over-compression.

Molecular mechanics/Poisson–Boltzmann surface area (MM/PBSA) calculations yielded a total binding free energy (Δ*G*_bind_) of −24.65 kcal/mol. This highly negative value signifies that the ligand–protein binding is thermodynamically favorable and characterized by a strong affinity. Energy decomposition analysis indicated that van der Waals interactions (Δ*E*_vdW_ = −36.05 kcal/mol) act as the primary driving force for binding, reflecting a high degree of shape complementarity between the ligand and the binding pocket. Per-residue energy contribution analysis further confirmed that hotspot residues ARG580, TYR583, PHE174, and LYS173 synergistically anchor OV16 within the TGM2 binding pocket through strong van der Waals forces and hydrogen bonding (Figure 2J).

In summary, the 100 ns MD simulation consistently demonstrated across multiple metrics that OV16 is a potent and stable binder of TGM2. The protein–ligand complex exhibits high structural stability, and OV16 achieves high-affinity binding through a persistent network of hydrogen bonds and van der Waals interactions. These findings provide a solid theoretical foundation for understanding the mechanism of action of OV16 and will guide subsequent structure-based drug optimization efforts.

### 2.3. The CETSA Identified TGM2 as a Potential Target of OV16

**Figure 3:**
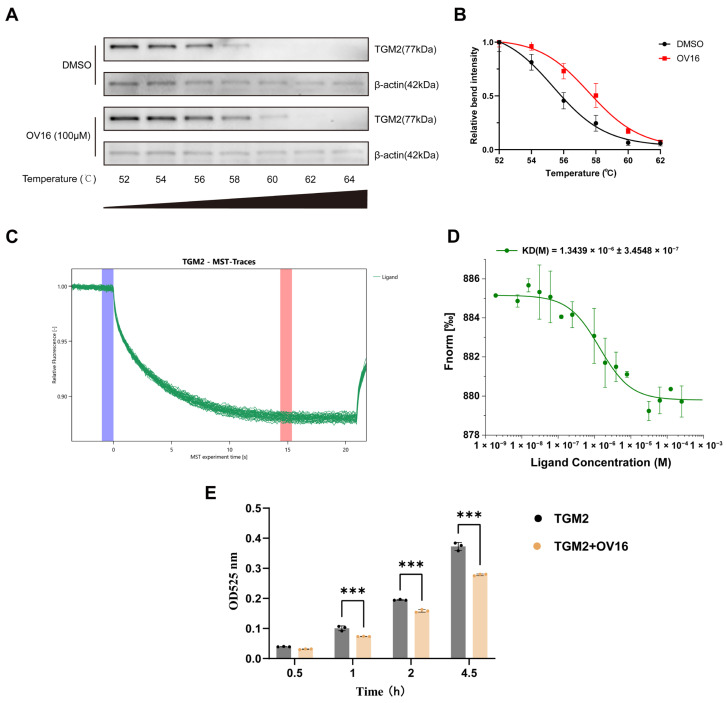
TGM2 is the target protein of OV16. (**A**) OV16 promotes the degradation of TGM2 (*n* = 3); (**B**) the fitting curve of CETSA; (**C**) MST trace line of the binding ability of OV16 and TGM2 in the microcalorimetric oscillation experiment (*n* = 3), each green curve corresponds to real-time fluorescence readings of one capillary, purple shows baseline average F0, and red is the averaged F1 across the fitting period.; (**D**) the fitted graph of Fnorm in the MST experiment; (**E**) the effect of OV16 on the transglutaminase activity of TGM2 (*n* = 3). *** *p* < 0.001.

To further validate the molecular targets of OV16, cell thermal shift assay (CETSA) was employed to identify potential target proteins directly binding to OV16 in intestinal epithelial cells. HIEC-6 cells treated with 100 μM OV16 were heated at temperatures of 52, 54, 56, 58, 60, 62, and 64 °C respectively. Western blot analysis revealed that OV16 protects TGM2 from heat-induced degradation, and CETSA results confirm that OV16 enhanced the thermal stability of TGM2 (Figure 3A,B).

### 2.4. MST Experimental Analysis of the Interaction Between OV16 and TGM2

Quantitative analysis of the binding affinity between TGM2 and OV16 was performed using microscale thermophoresis (MST). The MST technique directly measures the binding constant (Kd value) between drugs and targets with high sensitivity (nanomolar level) and minimal sample consumption by detecting changes in the migration rate of fluorescently labeled target molecules under a temperature gradient. MST results revealed that the migration rate of TGM2 protein increased with rising OV16 concentrations, exhibiting an inverted S-shaped curve with distinct upper and lower plateaus. This concentration-dependent pattern indicated specific binding with an affinity of 1.34 µM (Figure 3C,D). This binding strength is classified as high-affinity in protein–small molecule interactions. Such high affinity reduces potential adverse reactions and diminished therapeutic efficacy caused by drug interactions, allows for lower dosages, and minimizes effects on non-target molecules.

### 2.5. The Effect of OV16 on the Transglutaminase Activity of TGM2

Transglutaminase activity can be quantitatively determined by measuring the characteristic absorbance value at 525 nm of the product, l-glutamyl monohydroxamate, generated from the reaction of its substrate Z-Gln-Gly. This experiment examined whether compound OV16 affects TGM2 transglutaminase activity by monitoring the reaction system between TGM2 and its substrate Z-Gln-Gly. The results showed that during the 0.5–4.5 h reaction period, the absorbance increase rate in the OV16-treated group was significantly lower than that in the control group, with the difference exhibiting a time-dependent expansion (Figure 3E). This phenomenon suggests that OV16 binds to TGM2 and inhibits its transglutaminase function.

### 2.6. The Regulation of TGM2 Expression in Post-Radiation Cells by OV16

**Figure 4:**
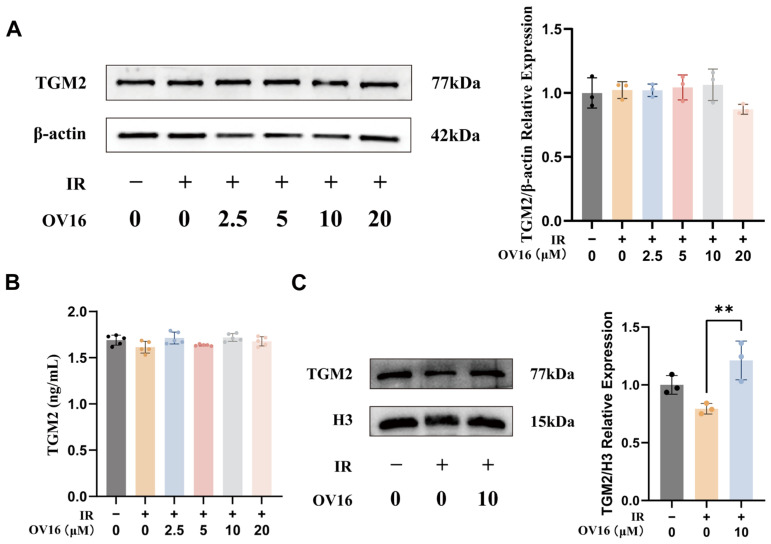
OV16 regulates the expression of TGM2 protein in HIEC-6 after radiation. (**A**) The effect of varying concentrations of OV16 on the TGM2 protein in cell lysates (*n* = 3); (**B**) the effect of varying concentrations of OV16 on the TGM2 protein in cell culture supernatants (*n* = 5); (**C**) the effect of OV16 on the TGM2 protein in the nucleus (*n* = 3). ** *p* < 0.01.

To investigate the effect of OV16 on TGM2 protein expression, HIEC-6 cells were treated with different concentrations of OV16 (0, 2.5, 5, 10, 20 μM) and irradiated with 15 Gy ^60^Co γ-rays. Western blot and ELISA analyses of TGM2 expression in cell lysates and culture supernatants revealed no dose-dependent changes in TGM2 protein levels, with no significant differences observed (Figure 4A,B). Subsequent nuclear fractionation and analysis revealed that OV16 increased nuclear TGM2 protein levels (Figure 4C), indicating its regulatory role in nuclear TGM2 expression.

### 2.7. The Increase in TGM2 Protein Helps Alleviate the Radiation Damage of HIEC-6 After Irradiation

**Figure 5:**
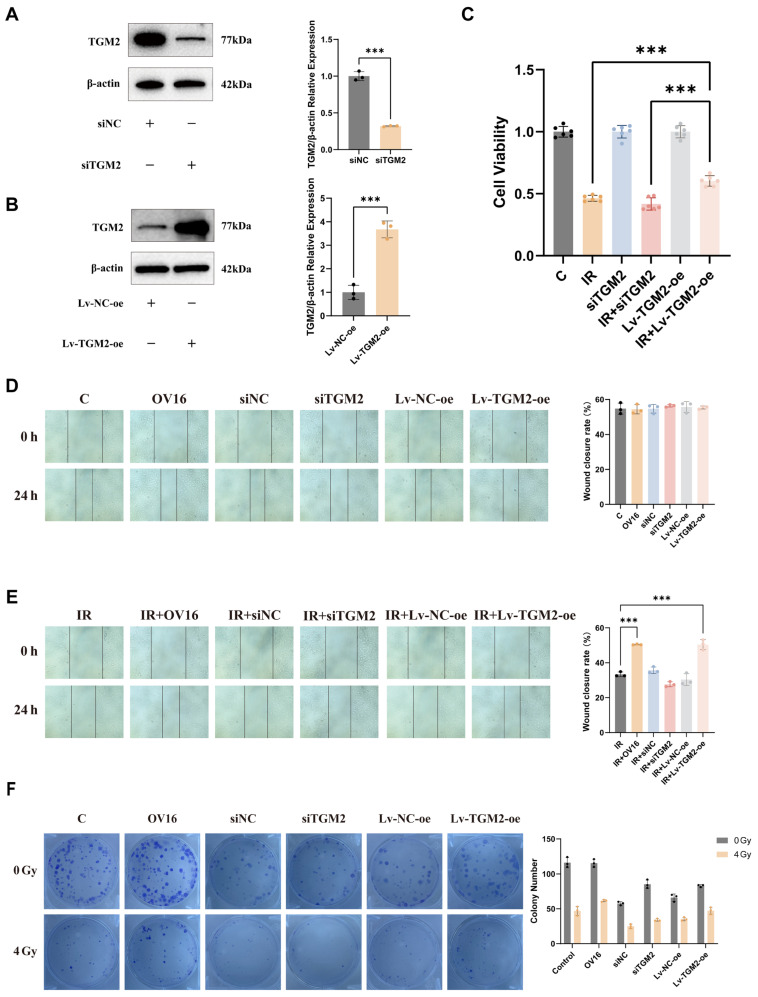
Elevated TGM2 protein helps alleviate the radiation-induced damage in HIEC-6 after irradiation. (**A**) Efficiency of protein knockdown after siTGM2 transfection (*n* = 3); (**B**) efficiency of protein overexpression after Lv-TGM2-oe infection (*n* = 3); (**C**) effect of TGM2 on cell proliferation after irradiation; (**D**) wound healing experiment of cells under normal conditions (*n* = 6); (**E**) wound healing experiment of cells after irradiation (*n* = 3); (**F**) the effect of TGM2 on cell colony formation (*n* = 3). *** *p* < 0.001.

To further validate the protective effect of TGM2 on irradiated HIE C-6 cells, TGM2 was knocked down via siRNA transfection and overexpressed via lentiviral infection. Western blot analysis confirmed the efficiency of TGM2 knockdown and overexpression (Figure 5A,B). CCK-8 assay data demonstrated that increased TGM2 expression in HIEC-6 cells enhanced their radiation resistance (Figure 5C). Subsequently, wound closure assay was assessed using the wound healing assay. Under non-ionizing radiation conditions, no significant differences in migration rates were observed among the groups at 24 h post-wounding (Figure 5D). However, under ionizing radiation conditions, observation at 24 h post-wounding revealed that the migration rates of cells in the OV16-treated group and the Lv-TGM2-oe group were higher than those in the other treatment groups, with extremely significant differences (Figure 5E). This indicates that TGM2 overexpression maintains the migratory capacity of HIEC-6 cells after irradiation, thereby promoting the reparative migration of epithelial cells and alleviating RIII. The results of the colony formation assay showed that under non-ionizing radiation conditions, TGM2 knockdown had already affected the growth ability of HIEC-6 cells. Following irradiation, both the number of colonies formed and the colony size in the siTGM2 group were further reduced. In the Lv-TGM2-oe group, the colony size increased after irradiation, but did not reach the level observed in the OV16-treated group (Figure 5F). These findings indicate that TGM2 overexpression partially preserves the proliferative potential of irradiated cells and enhances colony formation, thus conferring protection against RIII.

### 2.8. TGM2 Alleviates IR-Induced DSBs in HIEC-6

**Figure 6:**
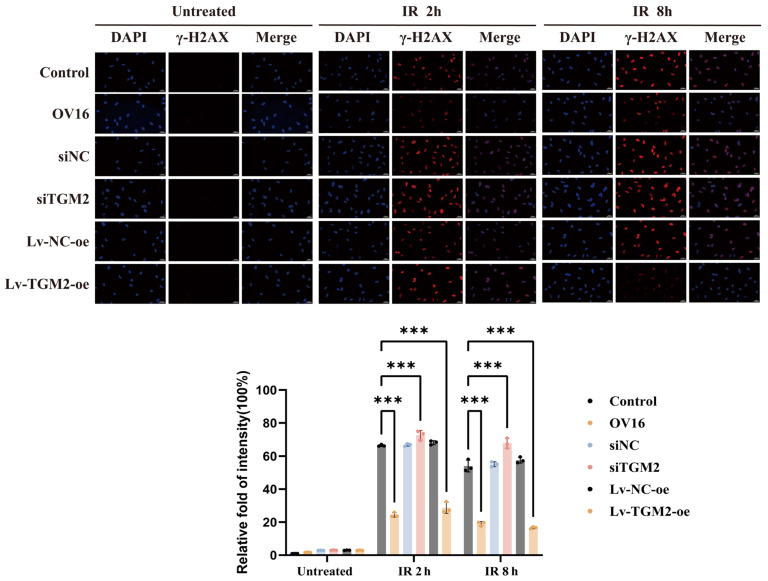
Fluorescence intensity of γ-H2AX in HIEC-6 after irradiation (*n* = 3). *** *p* < 0.001.

As a key biomarker of the DNA damage response, γ-H2AX specifically characterizes radiation-induced DSBs. Therefore, we investigated whether TGM2 contributes to mitigating radiation-induced DNA damage in HIEC-6 cells. As shown in Figure 6, after 8 h of irradiation, the fluorescence intensity of γ-H2AX in the Lv-TGM2-oe group exhibited a decreasing trend and was lower than that in other treatment groups. This result indicates that TGM2 helps mitigate radiation-induced DNA damage, thereby reducing cellular radiosensitivity and ultimately exerting an anti-RIII effect.

## 3. Discussion

This study first demonstrates that TGM2 confers radiation resistance to HIEC-6 cells under IR conditions by mediating DNA double-strand breaks (DSBs) repair. Although our enzyme activity experiments indicate that OV16 inhibits the transglutaminase activity of TGM2 to a certain extent, combined with the existing research results, during the DNA damage repair process, the binding pocket between OV16 and TGM2 does not involve THR162, TRP241, or other key residues that mediate TGM2 nuclear translocation and TGM2–TOPOIIα interaction [10]. Therefore, the binding of OV16 will not block the phosphorylation site THR162 or the key interface TRP241 of TGM2, thereby completely retaining the DNA repair-related functions of TGM2. This finding not only expands the functional understanding of TGM2 but also identifies a novel therapeutic target for RIII prevention. Current research on TGM2 and radiation response primarily focuses on tumor cells, with limited exploration in normal cells. Its effects exhibit significant cell specificity, and its regulatory mechanisms are unique, necessitating further investigation [9,10,11,24,25,26,27,28,29,30,31]. This study confirms TGM2’s radiation-protective role in HIEC-6 cells, filling a gap in the related research and offering a new direction for addressing this radiotherapy complication.

Subcellular localization is fundamental to TGM2 function, with nuclear translocation being critical for mediating radiation resistance. Under physiological conditions, TGM2 is primarily localized in the cytoplasm, with only 5–7% present in the nucleus [32]. TGM2 also has nuclear localization signals (NLS) and nuclear export signals (NES), which makes it possible to shuttle between the cytoplasm and the nucleus [33]. Stress like IR induce significant nuclear translocation, a feature confirmed in multiple tumor cell lines, suggesting this is a conserved event in TGM2’s radiation response. In contrast to tumor cells, this study found that TGM2 protein expression in the nuclei of irradiated HIEC-6 cells decreased. OV16 intervention restored its nuclear accumulation, suggesting that OV16 induces TGM2 nuclear translocation to exert anti-RIII effects. This suggests OV16 may exert its anti-RIII effects by promoting TGM2 nuclear translocation or stabilizing nuclear TGM2 protein. Therefore, the above results suggest differences in TGM2 nuclear accumulation dynamics in different cells, implying that its nuclear function exhibits temporal specificity. Nuclear translocation serves as a prerequisite for its nuclear function, laying the groundwork for subsequent mechanism research.

Furthermore, there are studies that have confirmed whether TGM2 can promote the differentiation and migration of bone marrow mesenchymal stem cells (BMSCs) by activating the Wnt/β-catenin signaling pathway, and enhance the wound healing ability of BMSCs [34]. This is consistent with the results of this study, where overexpression of TGM2 can to some extent enhance the migration ability of HIEC-6 after irradiation. This further validates that TGM2 has the function of promoting cell migration in normal cells.

Enhanced DNA damage repair capacity is a core mechanism underlying radiation resistance in tumor cells. This study and existing evidence indicate that TGM2 plays a key regulatory role in DSBs repair, with its mode of action exhibiting cell specificity. In Glioblastoma multiforme (GBM) cells, nuclear-localized TGM2 binds to p53 and promotes its degradation, enhancing radiation resistance [7]. In cervical cancer cells, TGM2 regulates radiosensitivity through ZNF domain protein (POGZ)-mediated cervical cancer DSBs repair, and the phenotype of TGM2 knockdown is consistent with the effect of POGZ deletion [8]. The mechanism in lung cancer cells is more complex: TGM2 interacts with TOPOIIα to regulate its activity, and its knockdown reduces NHEJ-associated protein levels, suggesting it may simultaneously regulate both HR and NHEJ pathways to modulate radiation resistance [10].

Targeting TGM2 inhibition represents a potential strategy for enhancing tumor radiotherapy efficacy, with multiple intervention approaches showing clinical promise. However, its mechanism of action is highly cell-specific, and there are significant differences in the regulatory pathways of different cells. Research on TGM2 in normal cells remains scarce, and future development must validate its effects on normal tissues, clarify functional differences between normal and diseased cells, and balance therapeutic efficacy with safety. In summary, TGM2 plays a key role in cellular radiation responses through nuclear translocation, protein interactions, and regulation of DSBs repair pathways. Despite its heterogeneous functional patterns and limited studies in normal cells, its core functions in promoting repair and modulating apoptosis are conserved, making it a promising target for enhancing radiotherapy efficacy and preventing RIII.

## 4. Materials and Methods

### 4.1. Drug Affinity Responsive Target Stability

Two dishes of cells were separately added to immunoprecipitation lysis buffer (20118ES60, Yeasen, Shanghai, China), scraped and collected in centrifuge tubes and lysed for 30 min on ice. The supernatant was collected by centrifugation and treated with protease. Each group was treated with 100 μM of OV16 and an equal volume of DMSO (D3855, Innochem, Beijing, China) for 20 min. Protease diluted with the prepared TNC solution was added and incubated for 15 min. Protein loading buffer (FD006, Fdbio Science, Hangzhou, China) containing protease inhibitors (IP0280, Solarbio, Beijing, China) was added and denatured by heating at 99 °C for 8 min. After the samples were separated by SDS-PAGE, the gels were subjected to mass spectrometry analysis for further evaluation.

### 4.2. Molecular Docking

The protein structure of TGM2 (PDB ID: 6A8P) was obtained from the PDB database. Prepare the ligand and protein files using the AutoDock tool (4.0). Optimize the protein by removing water molecules, adding hydrogen atoms and adding Geister charges, and save the protein and ligand files in PDBQT format. Calculate the protein–ligand grid map around the active site of the protein molecule. Molecular docking analysis was conducted via AutoDock Vina, and the output file generated contained theoretical binding affinities. Finally, the docking results were visualized using PyMol (3.1).

### 4.3. Molecular Dynamics

Molecular dynamics simulations of the complex of TGM2 and OV16 were conducted using GROMACS (version 2022) [35].The gmx_pdb2gmx command was used to generate the coordinate and topology files of the complex under global conditions, with the Charmm36 force field and SPC water environment selected as the simulation environment. To enhance computational efficiency, a dodecahedral box was chosen to be established in this experiment to confine the movement of the protein and ligand. The distance between the protein and the edge of the box was set at 1.0 nm to prevent the protein from escaping the confined box and thus disrupting the entire equilibrium system. Fill the dodecahedral box with an appropriate amount of SPC216 water solvent, and then add an appropriate amount of sodium ions or chloride ions to the system to make it electrically neutral. As the environment of the protein and ligand has changed, the simulation environment needs to be energy minimized to ensure the structure of the protein is relatively stable. In this experiment, the steepest descent strategy is selected for energy minimization. Since the intermolecular forces may change after the addition of the solvent, which could lead to alterations in the protein’s position and thus affect the stability of the system, the NVT and NPT commands are chosen to equilibrate the system. In the NVT pre-equilibration, the gas pressure is increased to 1 bar, and in the NPT pre-equilibration, the temperature is set at 310 K. After the protein’s positional constraints were completed, the simulated protein and ligand system reached a relatively stable equilibrium state, and then a 100 ns molecular dynamics simulation was conducted.

### 4.4. Cell Culture and γ-Irradiation

HIEC-6 derived from CTCC was cultured in Opti-MEM I Reduced Serum Medium, GlutaMAX Supplement (51985034, Thermo Scientific, Waltham, MA, USA) containing 4% FBS, 1% penicillin–streptomycin and 10 ng/mL EGF (C029, NOVOPROTEIN, Suzhou, China). The cells were maintained in a humidified atmosphere at 37 °C and 5% CO_2_ to ensure optimal growth conditions. Cell irradiation was performed using a ^60^Co γ-ray irradiation facility at the Academy of Military Medical Sciences (Beijing, China). Cells were exposed to a total dose of 15 Gy, with a dose rate ranging from 153.04 to 157.34 R/min, and the corresponding irradiation time ranged from 9 min 16 s to 9 min 56 s. Samples were placed 1.5 m away from the ^60^Co source to ensure homogeneous exposure.

### 4.5. Cell Thermal Shift Assay

HIEC-6 was treated with 100 μM of OV16 and an equal volume of DMSO for 24 h. The cells were then digested, centrifuged, and the supernatant was discarded. The cells were resuspended in PBS containing protease inhibitors and divided into equal parts. They were heated in a metal bath at a temperature gradient for 3 min and then placed on ice to cool. The cells were subjected to three cycles of freezing and thawing in liquid nitrogen and centrifuged again. The supernatant was collected and mixed with 5 × loading buffer, then boiled at 99 °C for 8 min. The target protein was analyzed by Western blot.

### 4.6. Microscale Thermophoresis Technology

The target protein TGM2 with His tag was labeled with RED-tris-NTA second-generation dye at room temperature in the dark for 30 min, and then the supernatant was collected after centrifugation. Take a small amount of the labeled protein, dilute it and load it into the capillary. Then, detect the fluorescence intensity and distribution in the instrument to confirm that there is no aggregation or adsorption, and optimize the detection parameters. Perform a 16-tube 2-fold serial dilution of OV16. Add an equal volume of labeled protein to each tube, mix well and incubate at room temperature for 15 min. Aspirate each concentration sample into the capillary tube in sequence and place it on the instrument tray. Start the detection. The infrared laser locally heats the capillary to form a temperature gradient, and monitor the change in fluorescence intensity over time. The instrument records the fluorescence signals at each concentration, fits the curve and calculates the affinity constant (KD value).

### 4.7. Determination of Transglutaminase Activity

According to the previous study [36], to investigate the effect of OV16 on the transglutaminase activity of TGM2, the absorbance was measured at a wavelength of 525 nm using an enzyme analyzer (A51119600C, Thermo Scientific, Waltham, MA, USA).

### 4.8. Western Blot

Proteins were separated by SDS-PAGE (PG212, Epizyme, Shanghai, China) and transferred onto a PVDF membrane (IPVH00010, Merck Millipore, Darmstadt, Germany). The membrane was then blocked with a protein-free rapid blocking solution (PS108P, EpiZyme, China) for 0.5 h, and then incubated with the specific primary antibody overnight. Subsequently, the membrane was incubated with the corresponding horseradish peroxidase (HRP)-labeled secondary antibody. Chemiluminescence detection was performed using the chemiluminescence detection reagent (36208ES76, Yeasen, Shanghai, China) with the chemiluminescence imaging system (ChemiDoc, Bio-Rad, Hercules, CA, USA), and the results were analyzed using Image J. The antibodies used were Transglutaminase 2 (ab2386, abcam, Cambridge, UK), β-actin (TT0022M, Abmart, Shanghai, China), Goat Anti-Mouse IgG(H+L) (LF101, Epizyme, Shanghai, China).

### 4.9. Enzyme-Linked Immunosorbent Assay

Cell supernatants were collected and the levels of TGM2 in the cell supernatants were detected using a Human Tissue Transglutaminase (TGM2) ELISA Kit (E-EL-H2336, Elabscience, Wuhan, China). The standard curve was established and samples were measured according to the specific operation manual. Finally, the optical density (OD value) of each well was measured at a wavelength of 450 nm using a microplate reader.

### 4.10. Nuclear and Cytoplasmic Protein Separation and Extraction

According to the manufacturer’s instructions, nuclear and cytoplasmic proteins were isolated using the Nuclear and Cyto-plasmic Protein Extraction Kit (PK10014, Proteintech, Wuhan, China).

### 4.11. Lentivirus Overexpression

The *TGM2* gene overexpression lentivirus and its negative control group virus used in this study were synthesized, constructed and packaged by Hunan Fenghui Biotechnology Co., Ltd. After adjusting the cell density to 2000 cells per well, the cells were seeded into 24-well culture plates and incubated in the incubator for 24 h. The volume of lentivirus required for infecting cells was calculated based on MOI = 200. Polybrene (BL628A, biosharp, Hefei, China) at a final concentration of 2 μg/mL was added to assist infection. After 24 h of infection, fresh culture medium was replaced for continued culture. The infection efficiency was observed under a fluorescence microscope after 72 h. The infected cells were further selected and expanded using 2 μg/mL puromycin (P8230, Solarbio, Beijing, China). Subsequently, the cells were collected for Western blot detection.

### 4.12. Silencing of TGM2 by Small Interfering RNA

Silencing of TGM2 by small interfering RNA (siRNA) interference plasmids were designed and synthesized by Hunan Fenghui Biotechnology Co., Ltd. siTGM2 was transfected into HIEC-6 using CALNP™ RNAi transfection reagent (DN001, D-Nano, Beijing, China). After 48 h, the cells were harvested for TGM2 expression analysis.

### 4.13. Cell Viability Assay

Cells were seeded in 96-well plates at a density of 5 × 10^3^ cells per well and cultured. After 96 h, 10 μL of CCK-8 (MA0218, Meilunbio, Dalian, China) was added to each well and incubated at 37 °C in 5% CO_2_ for 2 h. The absorbance at 450 nm was measured using a microplate reader.

### 4.14. Wound Closure Assay

Cells in the logarithmic growth phase were digested, centrifuged, resuspended and counted. After adjusting the cell density to 35 × 10^4^ cells per well, they were inoculated into 6-well culture plates. When the cells grow to approximately 90% confluence, use a 200 μL pipette tip to make parallel vertical lines in each well. Wash two to three times with PBS solution and continue to culture with serum-free medium. At 0 h and 24 h, photographs were taken through a microscope for observation, and the cell closure rate was measured and calculated to analyze the migration ability. The calculation formula for the cell migration rate is (0 h width–24 h width)/0 h width × 100%.

### 4.15. Colony Formation Assay

Cells were seeded in 6-well plates at a density of 5 × 10^3^ cells per well and cultured for 24 h. After drug treatment, they were exposed to ^60^Co γ-rays at doses of 0 Gy and 4 Gy. After 14 days of culture, the cells were fixed with 4% methanol for 30 min and then stained with crystal violet (C0121, Beyotime, Shanghai, China) for 30 min. After washing, the number of colonies was compared by taking pictures.

### 4.16. Immunofluorescence Assay for DNA Damage

Cells were seeded in 6-well plates, and different experimental groups were set up according to specific treatment conditions. According to the manufacturer’s instructions, DNA damage in cells was evaluated using the γ-H2AX immunofluorescence kit (C2036S, Beyotime, Shanghai, China). Observations and photography were conducted under a fluorescence microscope.

### 4.17. Statistical Analysis

Statistical analysis and graphing were performed using SPSS 27.0 and GraphPad Prism 10.0 software. Each experiment was independently repeated three times. All data are presented as the mean ± standard deviation (Mean ± SD). Comparisons between two groups were conducted using Student’s *t*-test, while comparisons among multiple groups were performed using analysis of variance (ANOVA). Statistical significance was set at * *p* < 0.05, ** *p* < 0.01, *** *p* < 0.001.

## 5. Conclusions

This study confirms that TGM2 is one of the target proteins of OV16, and OV16 can regulate the upregulation of TGM2 protein expression in the nuclei of HIEC-6 cells after γ-ray irradiation. Overexpression of TGM2 significantly enhances the proliferation activity and post-injury repair migration capacity of HIEC-6 cells while suppressing the generation of γ-H2AX, a specific marker of DNA double-strand breaks. These findings not only clarify the potential application value of OV16 as a preventive drug against RIII, but also lay an experimental foundation for targeting TGM2 in the prevention and treatment of RIII.

## Figures and Tables

**Table 1:** Top 10 proteins by DARTS comprehensive ranking.

Symbol	TGM2	ACO1	ATAD3B	CUL3	PTGR1	NT5DC2	BCS1L	GMPPB	NOP16	GMFG
Master	Master Protein	Master Protein	Master Protein	Master Protein	Master Protein	Master Protein	Master Protein	Master Protein	Master Protein	Master Protein
Accession	P21980	P21399	Q5T9A4	Q13618	Q14914	Q9H857	Q9Y276	Q9Y5P6	Q9Y3C1	O60234
Description	Protein-glutamine gamma-glutamyltransferase 2 OS = Homo sapiens OX = 9606 GN = TGM2 PE = 1 SV = 2	Cytoplasmic aconitate hydratase OS = Homo sapiens OX = 9606 GN = ACO1 PE = 1 SV = 3	ATPase family AAA domain-containing protein 3B OS = Homo sapiens OX = 9606 GN = ATAD3B PE = 1 SV = 1	Cullin-3 OS = Homo sapiens OX = 9606 GN = CUL3 PE = 1 SV = 2	Prostaglandin reductase 1 OS = Homo sapiens OX = 9606 GN = PTGR1 PE = 1 SV = 2	5′-nucleotidase domain-containing protein 2 OS = Homo sapiens OX = 9606 GN = NT5DC2 PE = 1 SV = 1	Mitochondrial chaperone BCS1 OS = Homo sapiens OX = 9606 GN = BCS1L PE = 1 SV = 1	Mannose-1-phosphate guanyltransferase beta OS = Homo sapiens OX = 9606 GN = GMPPB PE = 1 SV = 2	Nucleolar protein 16 OS = Homo sapiens OX = 9606 GN = NOP16 PE = 1 SV = 2	Glia maturation factor gamma OS = Homo sapiens OX = 9606 GN = GMFG PE = 1 SV = 1
Exp. q-Value: Combined	0	0	0	0	0	0	0	0	0	0
Sum PEP Score	298.526	190.963	94.563	88.823	85.648	61.427	51.387	45.527	25.057	16.376
Coverage [%]	39	44	35	32	40	37	32	29	40	35
# Peptides	29	28	21	19	12	14	9	8	6	4
# PSMs	439	221	146	96	82	67	35	53	22	19
# Unique Peptides	29	28	4	19	12	14	9	8	6	3
# AAs	687	889	648	768	329	520	419	360	178	142
MW [kDa]	77.3	98.3	72.5	88.9	35.8	60.7	47.5	39.8	21.2	16.8
calc. pI	5.22	6.68	9.2	8.48	8.29	6.77	8.5	6.61	9.94	5.26
Score Sequest HT: Sequest HT	1551.98	724.96	453.41	326.51	276.63	212.35	127.82	164.59	63.3	52.78
# Peptides (by Search Engine): Sequest HT	29	28	21	19	12	14	9	8	6	4
Fold Change	15.233	2.11243	21.479	2.34378	3.65018	2.03203	7.43837	5.05871	2.23505	2.17133

## Data Availability

The original data presented in this research are included in the article. Further inquiries can be directed to the corresponding author.

## References

[1] He D., Li Z., Wang M., Kong D., Guo W., Xia X., Li D., Zhou D. (2024). Metal-Organic-Framework-Based Sitagliptin-Release Platform for Multieffective Radiation-Induced Intestinal Injury Targeting Therapy and Intestinal Flora Protective Capabilities. J. Nanobiotechnol..

[2] Jackson S.P., Bartek J. (2009). The DNA-Damage Response in Human Biology and Disease. Nature.

[3] Lee K.-J., Saha J., Sun J., Fattah K.R., Wang S.-C., Jakob B., Chi L., Wang S.-Y., Taucher-Scholz G., Davis A.J. (2016). Phosphorylation of Ku Dictates DNA Double-Strand Break (DSB) Repair Pathway Choice in S Phase. Nucleic Acids Res..

[4] Batenburg N.L., Walker J.R., Noordermeer S.M., Moatti N., Durocher D., Zhu X.-D. (2017). ATM and CDK2 Control Chromatin Remodeler CSB to Inhibit RIF1 in DSB Repair Pathway Choice. Nat. Commun..

[5] Kang Y.-J., Yan C.T. (2018). Regulation of DNA Repair in the Absence of Classical Non-Homologous End Joining. DNA Repair.

[6] Bao S., Wu Q., McLendon R.E., Hao Y., Shi Q., Hjelmeland A.B., Dewhirst M.W., Bigner D.D., Rich J.N. (2006). Glioma Stem Cells Promote Radioresistance by Preferential Activation of the DNA Damage Response. Nature.

[7] Sun C., Du Z., Yang W., Wang Q. (2025). Transglutaminase 2 Nuclear Localization Enhances Glioblastoma Radiation Resistance. Discov. Oncol..

[8] Chi Y., Dong P., Zhang N., Liu B., Wang Q., Cheng G. (2025). TGM2 Regulates Radiosensitivity via POGZ-Mediated Repair of DNA Double-Strand Breaks in Cervical Cancer. Cancer Sci..

[9] Wang X., Shi W., Li M., Xin Y., Jiang X. (2024). RSL3 Sensitizes Glioma Cells to Ionizing Radiation by Suppressing TGM2-Dependent DNA Damage Repair and Epithelial-Mesenchymal Transition. Redox Biol..

[10] Lei X., Cao K., Chen Y., Shen H., Liu Z., Qin H., Cai J., Gao F., Yang Y. (2021). Nuclear Transglutaminase 2 Interacts with Topoisomerase IIα to Promote DNA Damage Repair in Lung Cancer Cells. J. Exp. Clin. Cancer Res..

[11] Tucholski J. (2010). TG2 Protects Neuroblastoma Cells against DNA-Damage-Induced Stress, Suppresses P53 Activation. Amino Acids.

[12] Sima L.E., Matei D., Condello S. (2022). The Outside-In Journey of Tissue Transglutaminase in Cancer. Cells.

[13] Yang Z., Zhang X., Zhuo F., Liu T., Luo Q., Zheng Y., Li L., Yang H., Zhang Y., Wang Y. (2023). Allosteric Activation of Transglutaminase 2 via Inducing an “Open” Conformation for Osteoblast Differentiation. Adv. Sci..

[14] Mo C., Li H., Yan M., Xu S., Wu J., Li J., Yang X., Li Y., Yang J., Su X. (2024). Dopaminylation of Endothelial TPI1 Suppresses Ferroptotic Angiocrine Signals to Promote Lung Regeneration over Fibrosis. Cell Metab..

[15] Ientile R., Campisi A., Raciti G., Caccamo D., Currò M., Cannavò G., Li Volti G., Macaione S., Vanella A. (2003). Cystamine Inhibits Transglutaminase and Caspase-3 Cleavage in Glutamate-exposed Astroglial Cells. J. Neurosci. Res..

[16] Chen J., Ma J., Qi D., Wang Y., Sun X., Yang J., Sun W., Luan C., Shan Q., Liu W. (2024). Inhibition of Transglutaminase 2 Inhibits Ionizing Radiation-induced Cellular Senescence in Skin Keratinocytes in Vitro. IUBMB Life.

[17] Sang T. (2025). Study on the Mechanism of *Orychophragine* D Targeting IRP1pathway to Prevent Radiation-Induced Intestinal Injury. Master’s Thesis.

[18] Zhang G., Sang T., Chen X., Ge C., Li B., Tian Y., Li M., Liu S., Xia Z., Li H. (2023). *Orychophragine* D:A New 2-Piperazinone Fused 5-Azacytosine Type Alkaloid with Radioprotective Activity from the Seeds of *Orychophragmus violaceus*. Fitoterapia.

[19] Zhang G., Dong J., Liu S., Li B., Tian Y., Li M. (2023). Application of Orychophragine D in the Preparation of Drugs against Radiation Damage.

[20] Xiao F., Zhang G., Du L., Lu Y., Cheng X., Dong J., Wang L. (2022). Application of Orychophragine D in the Preparation of Drugs Inhibiting Ferroptosis of Small Intestinal Crypt Epithelial Cells.

[21] D’Eletto M., Rossin F., Fedorova O., Farrace M.G., Piacentini M. (2019). Transglutaminase Type 2 in the Regulation of Proteostasis. Biol. Chem..

[22] Kumar S., Donti T.R., Agnihotri N., Mehta K. (2014). Transglutaminase 2 Reprogramming of Glucose Metabolism in Mammary Epithelial Cells via Activation of Inflammatory Signaling Pathways. Int. J. Cancer.

[23] Li B., Antonyak M.A., Druso J.E., Cheng L., Nikitin A.Y., Cerione R.A. (2010). EGF Potentiated Oncogenesis Requires a Tissue Transglutaminase-Dependent Signaling Pathway Leading to Src Activation. Proc. Natl. Acad. Sci. USA.

[24] Zheng W., Chen Q., Liu H., Zeng L., Zhou Y., Liu X., Bai Y., Zhang J., Pan Y., Shao C. (2023). SDC1-Dependent TGM2 Determines Radiosensitivity in Glioblastoma by Coordinating EPG5-Mediated Fusion of Autophagosomes with Lysosomes. Autophagy.

[25] Huaying S., Dong Y., Chihong Z., Xiaoqian Q., Danying W., Jianguo F. (2016). Transglutaminase 2 Inhibitor KCC009 Induces P53-Independent Radiosensitization in Lung Adenocarcinoma Cells. Med. Sci. Monit..

[26] Ai L., Skehan R.R., Saydi J., Lin T., Brown K.D. (2012). Ataxia-Telangiectasia, Mutated (ATM)/Nuclear Factor κ Light Chain Enhancer of Activated B Cells (NFκB) Signaling Controls Basal and DNA Damage-Induced Transglutaminase 2 Expression. J. Biol. Chem..

[27] Aepler J., Wodtke J., Wodtke R., Haase-Kohn C., Löser R., Pietzsch J., Hauser S. (2022). The Role of Transglutaminase 2 in the Radioresistance of Melanoma Cells. Cells.

[28] Zeng L., Zheng W., Liu X., Zhou Y., Jin X., Xiao Y., Bai Y., Pan Y., Zhang J., Shao C. (2023). SDC1-TGM2-FLOT1-BHMT Complex Determines Radiosensitivity of Glioblastoma by Influencing the Fusion of Autophagosomes with Lysosomes. Theranostics.

[29] Mann A.P., Verma A., Sethi G., Manavathi B., Wang H., Fok J.Y., Kunnumakkara A.B., Kumar R., Aggarwal B.B., Mehta K. (2006). Overexpression of Tissue Transglutaminase Leads to Constitutive Activation of Nuclear Factor-κB in Cancer Cells: Delineation of a Novel Pathway. Cancer Res..

[30] Wang Q., Zhang Q., Wang X., Luo H., Du T., Wu L., Tan M., Chen Y., Wu X., Sun S. (2024). TGM2-Mediated Autophagy Contributes to the Radio-Resistance of Non-Small Cell Lung Cancer Stem-like Cells. Biomedicines.

[31] Cao L., Petrusca D.N., Satpathy M., Nakshatri H., Petrache I., Matei D. (2008). Tissue Transglutaminase Protects Epithelial Ovarian Cancer Cells from Cisplatin-Induced Apoptosis by Promoting Cell Survival Signaling. Carcinogenesis.

[32] Kuo T., Tatsukawa H., Kojima S. (2011). New Insights into the Functions and Localization of Nuclear Transglutaminase 2. FEBS J..

[33] Shrestha R., Tatsukawa H., Shrestha R., Ishibashi N., Matsuura T., Kagechika H., Kose S., Hitomi K., Imamoto N., Kojima S. (2015). Molecular Mechanism by Which Acyclic Retinoid Induces Nuclear Localization of Transglutaminase 2 in Human Hepatocellular Carcinoma Cells. Cell Death Dis..

[34] Liu F., Wu M., Wu X., Chen D., Xie M., Pan H. (2023). TGM2 Accelerates Migration and Differentiation of BMSCs by Activating Wnt/β-Catenin Signaling. J. Orthop. Surg. Res..

[35] Cui Y., He Z., Chen T., Ren X., Xu J., Zhang S., Peng T., Liu S., Wang L. (2024). Design, Synthesis, Biological Evaluation and in Silico Studies of Novel Quinoline Derivatives as Potential Radioprotective Molecules Targeting the TLR2 and P53 Pathways. Eur. J. Med. Chem..

[36] Roberts E., Frankel S. (1950). Gamma-Aminobutyric Acid in Brain: Its Formation from Glutamic Acid. J. Biol. Chem..

